# A Rare Case of Salivary Duct Carcinoma at the Tip of the Tongue

**DOI:** 10.7759/cureus.76917

**Published:** 2025-01-04

**Authors:** Tadashi Chida, Yoshihiro Morita, Ryuhei Yamada, Osamu Ishihara, Narikazu Uzawa

**Affiliations:** 1 Department of Oral and Maxillofacial Oncology and Surgery, Osaka University, Osaka, JPN; 2 Department of Oral and Maxillofacial Surgery, Osaka General Medical Center, Osaka, JPN

**Keywords:** high-grade malignant tumor, intraoperative rapid diagnosis, minor salivary gland, salivary duct carcinoma, tip of the tongue

## Abstract

Salivary duct carcinoma (SDC) originating from the minor salivary glands is extremely rare. We report a case of SDC arising from the minor salivary gland tissue at the tip of the tongue. A 67-year-old man presented with a complaint of a mass and pain in the tip of the tongue. Magnetic resonance imaging showed a high-signal area isolated from the anterior lingual gland at the tip of the tongue. Since fine-needle-aspiration-cytology failed to diagnose a benign or malignant tumor, a diagnosis of suspected SDC was made by intraoperative rapid diagnosis, and the tumor was resected. The histopathological diagnosis was SDC. SDC is a high-grade malignant tumor of the salivary gland that is prone to local recurrence, lymph node metastasis, and distant metastasis. However, these results apply only to parotid and submandibular gland primary tumors, which occur more frequently. The prognosis of SDC of minor salivary gland primary tumors is not still clearly understood. In this report, the patient with SDC from the minor salivary gland tissue at the tip of the tongue had no recurrence or metastasis 14 years after the surgery.

## Introduction

There have been few cases of malignant tumors starting near the tip of the tongue, while the majority of tongue cancers start along the tongue's edge [1]. To the best of our knowledge, the occurrence of salivary duct carcinoma (SDC) starting at the tip of the tongue has not been reported. Salivary duct malignancies that arise in the salivary glands are high-grade, locally aggressive malignancies with a tendency for regional and distant metastases [2]. The frequency of occurrence is approximately 1% of all salivary gland tumors, and the parotid gland is the most common site of occurrence, followed by the submandibular gland. SDC originating from the minor salivary glands is extremely rare [3]. Approximately 20% and 50% of tumors that affect the major and minor glands, respectively, are malignant [4]. SDC is diagnosed mainly based on hematoxylin and eosin (H-E) staining of the tumors and a combination of H-E and immunohistochemical tests, such as androgen receptor (AR) and HER2 protein staining. Histopathological diagnosis requires adequate biopsy material stained with H-E to uncover the full range of morphological and cytological characteristics to reduce the risk of misdiagnosis. We present a rare case of a patient with SDC of the anterior lingual gland who initially underwent fine-needle-aspiration-cytology (FNAC) and could not be diagnosed. Despite SDC being a malignant tumor known to have a very poor prognosis, this patient did not show recurrence or metastasis for a very long period of 14 years after surgery.

## Case presentation

A 69-year-old man presented to our department with a chief complaint of a mass and pain in his tongue. He had been experiencing pain in his tongue for a month; therefore, he visited our hospital. He had been aware of a mass on the apex of his tongue for a year prior to his visit to our hospital; however, the lesion was left untreated as it was asymptomatic. He had a history of hypertension and was taking antihypertensive medication. An elastic, hard mass with a diameter of 10 mm was found at the center of the tip of the tongue. The coated mucosa was normal, and there were no sensory or motor deficits. The physical condition of the patient was good; no abnormalities were noted in the neck.

**Figure 1:**
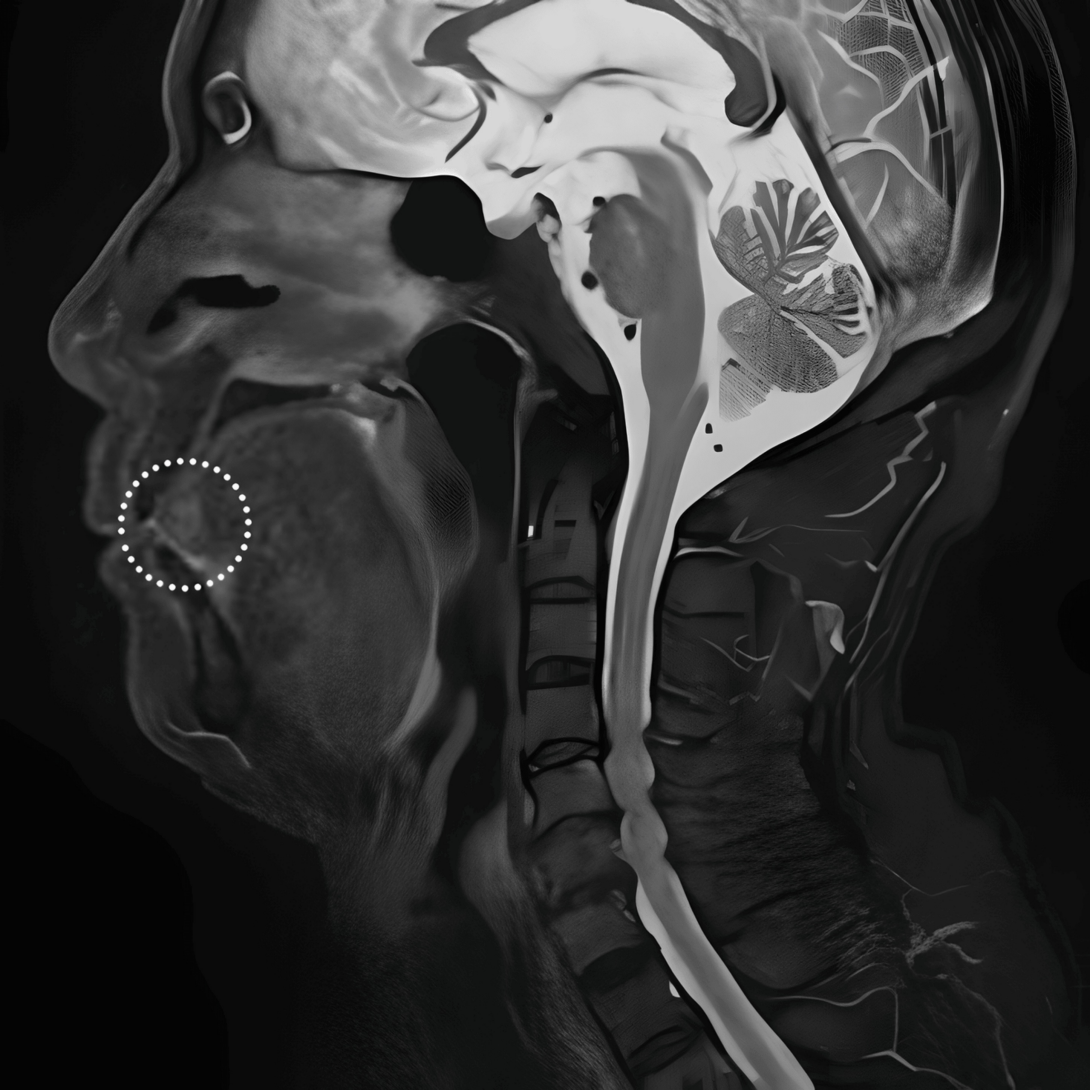
Magnetic resonance imaging The T2-weighted image shows a slightly high-signal area (dotted line) at the apex of the tongue.

Magnetic resonance imaging (MRI) revealed a high-signal region with a relatively clear border that was partially contiguous with the anterior lingual gland on T2-weighted images at the tip of the tongue (Figure 1).

**Figure 2:**
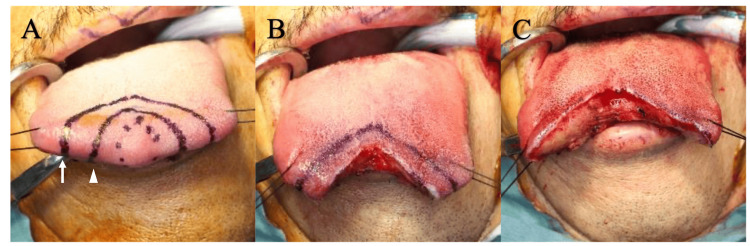
Surgical photos (A) A mass of approximately 10 mm length and diameter was found at the apex of the tongue. The resection line indicated by the arrowhead is a safety margin of approximately 3 mm from the mass, and the resection line indicated by the arrow is a safety margin of approximately 10 mm from the mass. (B) After resection with a safety margin of 3 mm. (C) After additional resection with a safety margin of 10 mm.

**Figure 3:**
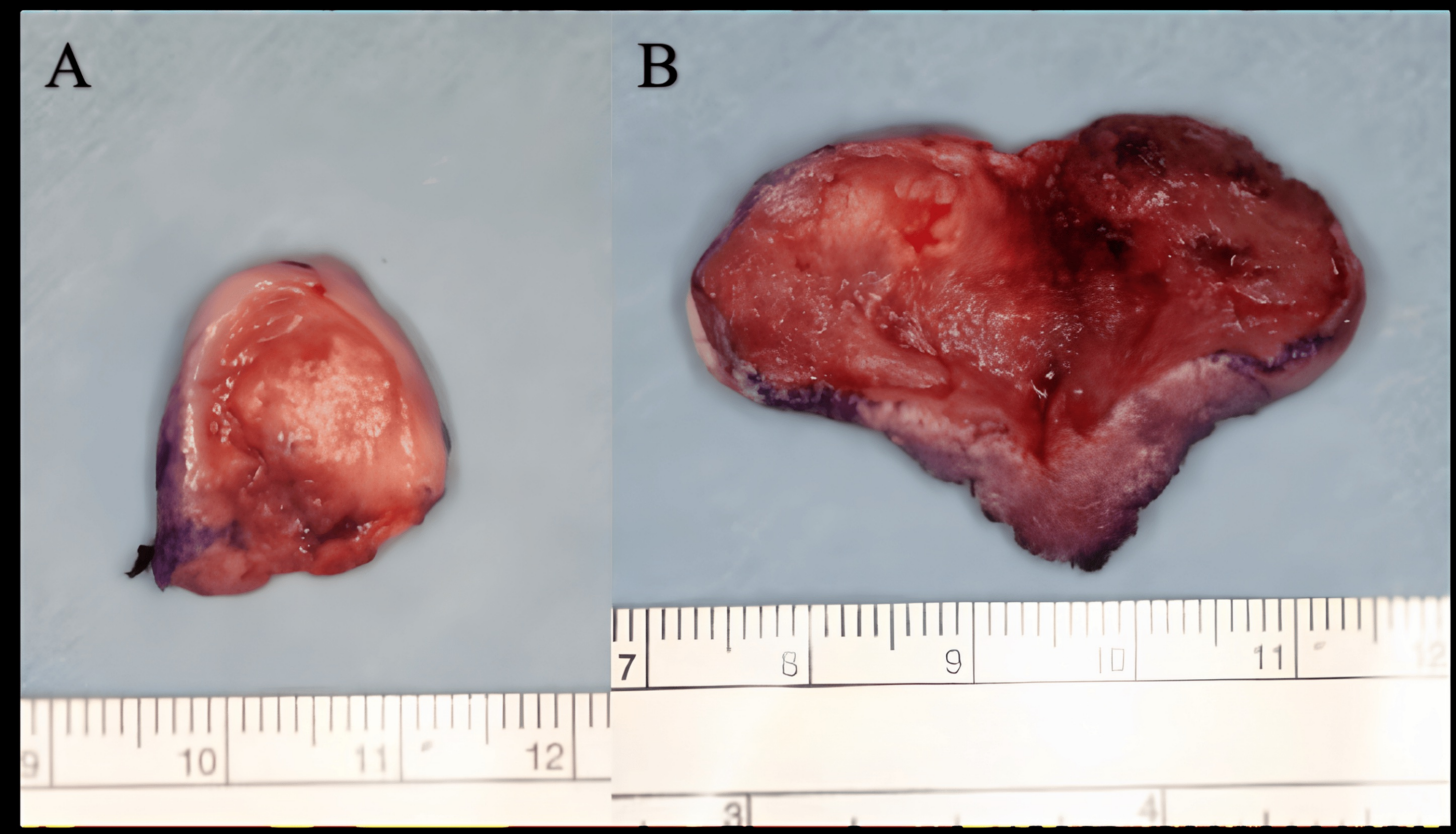
Resection specimen (A) Resection specimen with a safety margin of 3 mm. (B) Additional resection specimen with a safety margin of 10 mm.

FNAC performed for the clinical diagnosis of the lesion showed a few cells with a large naked nucleus; however, it did not lead to a differential diagnosis of a benign or malignant tumor. Therefore, tumor resection was planned at the same time as the operative rapid pathological diagnosis with a frozen section under general anesthesia. Since the preoperative examination did not show a malignant tumor, the safety margin was set as 3 mm and 10 mm from the tumor (Figure 2A). Tumor resection was performed initially with a safety margin of 3 mm (Figure 2B). After obtaining the intraoperative frozen section diagnosis of suspected SDC, an additional 7 mm was resected, and the defect was sutured (Figures 2C, 3A, 3B).

**Figure 4:**
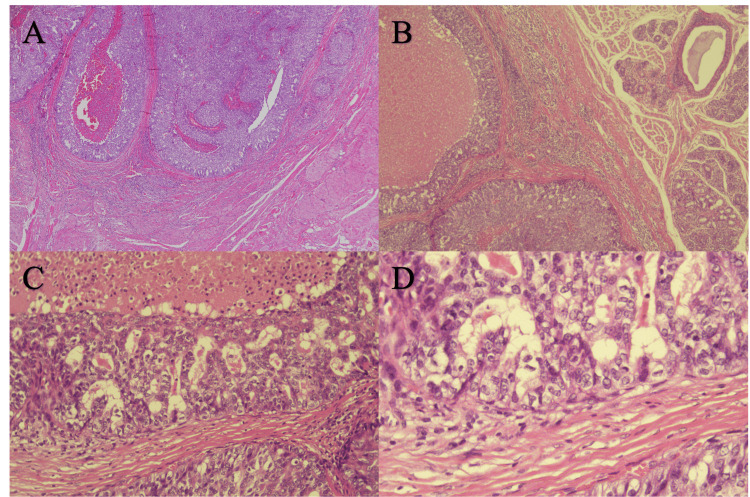
Histopathological findings Histological study of the excised lesion revealed the tumor invading toward the deep portion of the lingual muscle (A). Large atypical cells with eosinophilic cytoplasm form large and small foci, some of which show central necrosis known as comedo necrosis (A, B). Minor salivary glands were found beside the tumor nest (B). In higher magnification, there was comedo necrosis within the tumor nest, the roman-bridge architecture of the tumor nest, and fibrosis in the stroma (C). There is a lack the basal cell/myoepithelial cell suggesting that these tumor nests are invasive components. There was nuclear atypia in higher magnification (D). Hematoxylin and eosin stain (Magnification: A ×50, B ×100, C ×200, D ×400).

Histologically, large, atypical cells with eosinophilic cytoplasm formed large and small foci, some of which showed central necrosis known as comedo necrosis. A characteristic cribriform structure called a Roman bridge, an irregular glandular luminal formation, and cancer cells invading muscle and normal salivary glands were observed in the lesion. Moreover, there were no nodules or nodular scars suspected of pleomorphic adenoma and no extensive intraductal extension (Figures 4A-4D).

The SDC was diagnosed from these histological findings. Other differential diagnoses included mucoepidermoid carcinoma, polymorphous adenocarcinoma, adenocarcinoma, tumor with differentiation into myoepithelium and basal cells, and adenocarcinoma not otherwise specified (NOS). The lack of squamous differentiation differentiated it from mucoepidermoid carcinoma, and the large size and atypical nature of the tumor cells differentiated it from polymorphous adenocarcinoma. The tumor was completely resected, and the distance to the resection margin of the tumor was more than 8 mm.

**Figure 5:**
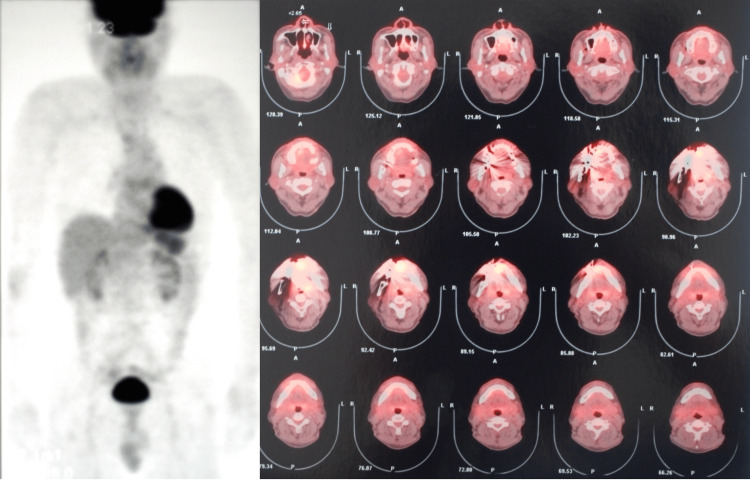
FDG-PET/CT scans performed after the postoperative definitive diagnosis showing no cervical lymph node or distant metastases FDG - fluorodeoxyglucose

The postoperative course of the patient was good. No cervical lymph node or distant metastases were found on and fluorodeoxyglucose (FDG)-PET/CT scans performed after the postoperative definitive diagnosis (Figure 5). Therefore, postoperative adjuvant treatment was not administered. No local recurrence, cervical lymph node metastasis, or distant metastasis was observed for 14 years after the surgery until the patient was urgently admitted to another hospital for a subdural hematoma and finally dead.

## Discussion

The lingual glands, which are minor salivary glands, are divided into three groups: the anterior lingual glands (Blandin Nuhn glands), located at the lower tip of the tongue; the posterior lingual glands, located at the base of the tongue and posterior to the lateral border of the tongue; and the Ebner's glands, located around the contour and lobular papillae. Goldblatt et al. analyzed 55 cases of primary minor salivary gland tumors of the tongue and reported that the majority were malignant (50 cases), more than 85% of the malignant tumors were located at the root of the tongue, and five benign tumors were myoepitheliomas, 80% of which (four cases) were located anterior to the central portion of the tongue [1]. Therefore, most salivary gland tumors involving the tongue are malignant and originate from the glands in the posterior aspect of the tongue, and malignant salivary gland tumors occurring in the tongue tip are very rare. We found four cases of carcinoma originating from the glands in the tongue tip over the last 10 years; however, only the present case and one other case were diagnosed as SDC (Table 1) [5-8].

**Table 1:** The list of carcinomas originating from the glands in the tongue tip over the last 10 years SDC: Salivary duct carcinoma, ACC: Adenoid cystic carcinoma, LGMC: Low-grade mucoepidermoid carcinoma

Reference	Year	Gender	Age	Size (mm)	Metastasis	Treatment	Pathological diagnosis	Follow-up (months)	Outcome
Kumar et al. [5]	2016	Male	60	50×30×20	－	Surgery	ACC	36	Alive
Anwer et al. [6]	2018	Male	52	Unknown	－	Surgery	SDC	13	Alive
Tang et al. [7]	2019	Male	43	23×19	－	Surgery	ACC	29	Alive
Shimpei et al. [8]	2021	Female	82	19×15	－	Surgery	LGMC	12	Alive
Present case	2022	Male	69	10×10×10	－	Surgery	SDC	168	Alive

SDC is a salivary gland carcinoma with a histopathological picture similar to ductal carcinoma of the mammary gland, first described by Kleinsasser et al. in 1968 [9]. SDC is a relatively rare tumor, accounting for approximately 1% of all salivary gland tumors [3]. SDC is frequently observed in 60-70-year-old individuals, with a male-to-female ratio of generally 6:4, and most cases are reported to be primary to the parotid or submandibular gland [10]. Therefore, SDC of the minor salivary glands is very rare [11]. SDC is a high-grade malignant tumor of the salivary gland that is prone to local recurrence, lymph node metastasis, and distant metastasis, with a reported five-year survival rate of 23.2% [12]. However, these results apply only to parotid and submandibular gland primary tumors, which occur more frequently; the prognosis of SDC of minor salivary gland primary tumors is not still clearly understood. Anwer et al. report 12 SDC cases. Among them, there are two cases of SDC derived from minor salivary glands (buccal mucosa and anterior tongue), both of which are described as alive, but the observation period is short, about one year, and the detail for prognosis is unknown [6].

Histopathologically, SDC shows a proliferative pattern of cribriform, papillary, and solid types with glandular tubular structure and is characterized by central necrosis known as comedo-necrosis. Immunohistochemistry may be positive for AR, HER2 protein, keratin, and epithelial membrane antigen (EMA) but negative for S-100 protein and myosin, which are positive in myoepithelial cell-derived tumors [1]. The present case was able to be diagnosed as SDC because of the typical findings of H-E staining. No additional immunostaining was performed, partly because the prognosis was good.

Early diagnosis is important because of its poor prognosis. FNAC is capable of diagnosing SDC in primary cases of the parotid and submandibular glands based on the presence of a papillary growth pattern and intranuclear vacuolar degeneration [13,14]. Despite performing FNAC and the small size of the tumor, malignancy could not be ruled out in the present case. However, we did not proceed with an incisional biopsy due to the prognostic impact of cell seeding associated with malignant tumors of the salivary glands. Moreover, since the tumor diameter was not very large (approximately 10 mm), tumor resection was performed based on the intraoperative frozen-section diagnosis. Since SDC was suspected as the frozen-section diagnosis, extended resection was performed subsequently.

There is no established treatment for SDC, and surgery is the first choice of treatment. Due to the poor prognosis of SDC, radiation therapy and chemotherapy are used as adjuvant therapy. However, there is no consensus on the content of chemotherapy, and the choice varies with each hospital. Recently, anti-androgen therapy and anti-HER-2 therapy for AR- and HER-2-positive SDC have been successful and are expected to improve prognosis [15-17].

Although SDC has a poor prognosis, most studies examined cases of major malignant salivary gland tumors that occur more frequently. In contrast, few studies have examined the prognosis of a large number of minor malignant salivary gland tumors. Urban et al. found that the prognosis of minor salivary gland malignant tumors was better than that of major salivary gland malignant tumors as they were often painful and detected at a relatively early stage [18]. Delgado et al. proposed the classification of low-grade SDC and high-grade SDC and noted that low-grade SDC does not show papillary growth patterns or comedo formation as histological features [19]. No recurrence or subsequent metastasis was observed, and the postoperative course was good, although there were histopathological findings of comedo formation, and the SDC was classified as high-grade. We speculate that this was due to the small size of the tumor, the lack of vascular invasion on histology, and the resection being performed with a sufficient safety margin. Since the site of origin of this lesion was the tip of the tongue, resection was not difficult, and the risk of recurrence was expected to be low. In addition, the tip of the tongue would have fewer large blood and lymphatic vessels than other parts of the tongue, which might reduce the risk of metastasis. However, there is still a need for additional investigation regarding this, but it will be difficult due to the small number of cases.

## Conclusions

This is a case of SDC that developed from the tissue of the small salivary glands at the tongue's tip. A diagnosis of suspected SDC was made by intraoperative rapid diagnosis, and the tumor was resected. The histopathological diagnosis was SDC. Although SDC has a poor prognosis, the patient had no recurrence or metastasis 14 years after the surgery. This may be due to the site of originating, the tip of the tongue, but it is still not based on the evidence.

## References

[1] Goldblatt LI, Ellis GL (1987). Salivary gland tumors of the tongue. Analysis of 55 new cases and review of the literature. Cancer.

[2] Johnston ML, Huang SH, Waldron JN (2016). Salivary duct carcinoma: treatment, outcomes, and patterns of failure. Head Neck.

[3] Seifert G (1997). Diagnosis and prognosis of salivary gland tumors. An interpretation of new revised WHO classification (Article in German). Mund Kiefer Gesichtschir.

[4] Lin HH, Limesand KH, Ann DK (2018). Current state of knowledge on salivary gland cancers. Crit Rev Oncog.

[5] Kumar S, Agarwal P, Nimmi V (2016). Adenoid cystic carcinoma: a rare late presentation of the mobile tongue. J Oral Biol Craniofac Res.

[6] Anwer AW, Faisal M, Adeel M (2018). Clinicopathological behavior and treatment-related outcome of rare salivary duct carcinoma: the Shaukat Khanum Memorial Cancer Hospital experience. Cureus.

[7] Tang X, Zhang C, Chen R, Zhou X, Zhang Y (2019). Treatment of adenoid cystic carcinoma of the mobile tongue with anterolateral thigh flap reconstruction: a case report. Medicine (Baltimore).

[8] Gotoh S, Nakasone T, Matayoshi A (2022). Mucoepidermoid carcinoma of the anterior lingual salivary gland: a rare case report. Mol Clin Oncol.

[9] Kleinsasser O, Klein HJ, Hübner G (1968). Salivary duct carcinoma. A group of salivary gland tumors analogous to mammary duct carcinoma (Article in German). Arch Klin Exp Ohren Nasen Kehlkopfheilkd.

[10] Han EJ, Mukdad LA, Alhiyari Y, Nakhla MN, Sajed DP, St John MA (2024). A 22-year single institution review of 119 cases of salivary duct carcinoma. Laryngoscope Investig Otolaryngol.

[11] Lewis JE, McKinney BC, Weiland LH, Ferreiro JA, Olsen KD (1996). Salivary duct carcinoma. Clinicopathologic and immunohistochemical review of 26 cases. Cancer.

[12] Jaehne M, Roeser K, Jaekel T, Schepers JD, Albert N, Löning T (2005). Clinical and immunohistologic typing of salivary duct carcinoma: a report of 50 cases. Cancer.

[13] Hughes JH, Volk EE, Wilbur DC (2005). Pitfalls in salivary gland fine-needle aspiration cytology: lessons from the College of American Pathologists interlaboratory comparison program in nongynecologic cytology. Arch Pathol Lab Med.

[14] Lin AC, Bhattacharyya N (2007). The utility of fine needle aspiration in parotid malignancy. Otolaryngol Head Neck Surg.

[15] Dalin MG, Watson PA, Ho AL, Morris LG (2017). Androgen receptor signaling in salivary gland cancer. Cancers (Basel).

[16] Jaspers HC, Verbist BM, Schoffelen R, Mattijssen V, Slootweg PJ, van der Graaf WT, van Herpen CM (2011). Androgen receptor-positive salivary duct carcinoma: a disease entity with promising new treatment options. J Clin Oncol.

[17] Soper MS, Iganej S, Thompson LD (2014). Definitive treatment of androgen receptor-positive salivary duct carcinoma with androgen deprivation therapy and external beam radiotherapy. Head Neck.

[18] Urban SD, Hall JM, Bentkover SH, Kadish SP (2002). Salivary duct carcinoma of minor salivary gland origin: report of a case involving the cavernous sinus. J Oral Maxillofac Surg.

[19] Delgado R, Vuitch F, Albores-Saavedra J (1993). Salivary duct carcinoma. Cancer.

